# Benchmarking Non-Targeted Metabolomics Using Yeast-Derived Libraries

**DOI:** 10.3390/metabo11030160

**Published:** 2021-03-10

**Authors:** Evelyn Rampler, Gerrit Hermann, Gerlinde Grabmann, Yasin El Abiead, Harald Schoeny, Christoph Baumgartinger, Thomas Köcher, Gunda Koellensperger

**Affiliations:** 1 Department of Analytical Chemistry, Faculty of Chemistry, University of Vienna, Währinger Str. 38, 1090 Vienna, Austria; evelyn.rampler@univie.ac.at (E.R.); gerrit.hermann@univie.ac.at (G.H.); yasin.el.abiead@univie.ac.at (Y.E.A.); harald.schoeny@univie.ac.at (H.S.); christoph.baumgartinger@univie.ac.at (C.B.); 2 Vienna Metabolomics Center (VIME), University of Vienna, Althanstraße 14, 1090 Vienna, Austria; 3 ISOtopic Solutions, Währinger Str. 38, 1090 Vienna, Austria; 4 Metabolomics Core Facility, Vienna BioCenter Core Facilities, Dr.-Bohr-Gasse 3, 1030 Vienna, Austria; gerlinde.grabmann@vbcf.ac.at (G.G.); Thomas.Koecher@vbcf.ac.at (T.K.)

**Keywords:** non-targeted metabolomics, quality control, yeast, metabolites, lipids, benchmarking, database, mass spectrometry, library

## Abstract

Non-targeted analysis by high-resolution mass spectrometry (HRMS) is an essential discovery tool in metabolomics. To date, standardization and validation remain a challenge. Community-wide accepted cost-effective benchmark materials are lacking. In this work, we propose yeast (*Pichia pastoris*) extracts derived from fully controlled fermentations for this purpose. We established an open-source metabolite library of >200 identified metabolites based on compound identification by accurate mass, matching retention times, and MS/MS, as well as a comprehensive literature search. The library includes metabolites from the classes of (1) organic acids and derivatives (2) nucleosides, nucleotides, and analogs, (3) lipids and lipid-like molecules, (4) organic oxygen compounds, (5) organoheterocyclic compounds, (6) organic nitrogen compounds, and (7) benzoids at expected concentrations ranges of sub-nM to µM. As yeast is a eukaryotic organism, key regulatory elements are highly conserved between yeast and all annotated metabolites were also reported in the human metabolome database (HMDB). Orthogonal state-of-the-art reversed-phase (RP-) and hydrophilic interaction chromatography mass spectrometry (HILIC-MS) non-targeted analysis and authentic standards revealed that 104 out of the 206 confirmed metabolites were reproducibly recovered and stable over the course of three years when stored at −80 °C. Overall, 67 out of these 104 metabolites were identified with comparably stable areas over all three yeast fermentation and are the ideal starting point for benchmarking experiments. The provided yeast benchmark material enabled not only to test for the chemical space and coverage upon method implementation and developments but also allowed in-house routines for instrumental performance tests. Transferring the quality control strategy of proteomics workflows based on the number of protein identification in HeLa extracts, metabolite IDs in the yeast benchmarking material can be used as metabolomics quality control. Finally, the benchmark material opens new avenues for batch-to-batch corrections in large-scale non-targeted metabolomics studies.

## 1. Introduction

Non-targeted analysis (NTA) by high-resolution mass spectrometry (HRMS) is a prime example of an innovative measurement practice and the key to discoveries in many applications, such as different “omics” areas [1], environmental protection [2], and food safety [3]. NTA will not replace targeted analysis but emerged as a complementing cost-effective discovery tool. Especially omics-scale research profits from the improved coverage and the lower costs of analysis provided by HRMS. The common challenge in all fields of application is to create the reliability of data and to find ways of harmonization, especially in large-scale multicenter studies [4,5,6]. As a matter of fact, validation practices and guidelines from the targeted analysis cannot be simply applied. Thus, validation in NTA is significantly less developed and defined. This fact, together with the wide acceptance of the NTA toolset, calls for new strategies of standardization, quality control measures, and metrics for evaluation. Joint efforts towards harmonized NTA protocols and definition of a minimum of quality requirements are of paramount importance in this endeavor.

Metabolomics is one of the key applications of NTA. The metabolomics standardization initiative (MSI) of the metabolomics society [7] has worked intensively on definitions and guidelines considering all steps of the NTA analytical process for many years. This includes defining the analytical task, sampling/analysis of data standards, data evaluation, and reporting [8,9]. NTA was addressed with regard to the annotation of metabolites and their relative quantification at all levels of analysis. MSI recommends that all researchers define the level of identification, a common name, and a structural code (e.g., InChI or SMILES) in their publications [10]. MSI also highlights the need to submit the data to open-access repositories, such as MetaboLights [11], to provide clarity of NTA data. The impact of this initiative on all levels of NTA was massive and significantly improved the harmonization in the field [5].

Reference materials meeting all stringent metrological criteria of full traceability are scarce in metabolomics and in life sciences in general. One reason might be the rather poor acceptance of reference materials as their extensive use in large-scale omics-type measurement campaigns increase costs significantly. In fact, the list of available reference materials for metabolomics is very short. Only very recently (June 2020), the first untargeted metabolomics study on a large scale basis comparing three pooled human plasma reference materials (Qstd3, 211 CHEAR, NIST1950) was reported [12]. In large-scale metabolomics studies, the concept of a pooled sample for quality control has gained worldwide acceptance allowing to correct for intra- and inter-batch variations and to accomplish MS/MS measurements required for annotation [5]. However, in multicenter studies, as envisioned in clinical metabolomics, it is not straightforward to produce pooled samples in sufficient amounts. Additionally, in many large-scale investigations (e.g., longitudinal clinical studies or population profiling), all samples are not available at the beginning of the analysis, so preparing a pooled sample is impossible.

In other omics fields, such as proteomics, the concept of affordable and easily accessible benchmark materials prevailed. HeLa cell extracts have become the gold standard for benchmarking instrument performance and proof-of-principle experiments upon the introduction of new analytical methods [13,14,15,16,17]. Many laboratories and instrument manufacturers resort to tryptic digests of protein extracts from HeLa cells to check performance [16], prove a method fit-for-purpose, benchmark new proteomics workflows [13], or show significant technological progress [18]. All metrics are established for quality control, protein identification numbers, and reported compound areas.

Currently, there is no such commonly accepted low-cost biological matrix material in metabolomics. In this work, we explore yeast as a potential benchmark material for metabolomics. Yeasts are industrially important cell factories, easy to cultivate in a short time and large populations using inexpensive media. Despite the phylogenetical distance, a number of key regulatory elements are highly conserved between yeast and humans [19]. In the field of metabolomics, ^13^C enriched yeast has become widely accepted as a resource for ^13^C internal standards. Controlled growth conditions of *Pichia pastoris* are ideal for producing ^13^C-labeled metabolites with efficiencies higher than 99%, leading to the simultaneous production of hundreds of biologically relevant labeled metabolites [20,21,22]. These highly enriched compounds enabled absolute quantification of a wide range of metabolites and lipids [20,23,24,25] as well as validation of new software tools for non-targeted data evaluation, such as the METLIN platform (using fragment matches of labeled and non-labeled metabolite pairs) [26].

In this work, we propose the use of ethanolic yeast extracts from *Pichia pastoris* in vivo fermentation as stable and low-cost controls for non-targeted metabolomics workflows. Evidently, a benchmark material cannot replace certified reference materials, but as in proteomics, it could play a significant role in facilitating the method development and validation. A first crucial step in this direction is our reported metabolite library.

## 2. Results

Over the recent past, yeast-based standards have seen a slow but increasing acceptance in the metabolomics community. Several laboratories resorted to *Pichia pastoris* ethanolic yeast extracts [27,28,29,30,31,32,33,34,35,36] primarily for ^13^C internal standardization. Only a few studies were related to instrument performance tests or method development in accordance with the here proposed application [30,35,36,37].

### 2.1. Metabolite Identification and Quality of the Proposed Benchmark Material

#### 2.1.1. Metabolites and Lipids Present in the Yeast Material

The yeast extract was produced in 3 different years (2017–2019) from controlled *Pichia pastoris* (Guillierm.) Phaff 1956 (*Komagataella phaffii Kurtzman*) [38] fermentation followed by quenching and boiling ethanol metabolite extraction, the state-of-the-art extraction procedure. One aliquot of yeast ethanolic extract contains 15 mg of dried cell extract originating from 20 mg yeast cell dry weight of approximately 2 billion cells (corresponding to the commercially available yeast extract). The metabolite inventory provided in this work is primarily based on data collected in two different laboratories (Köllensperger lab, University Vienna; Vienna BioCenter Core Facilities, Austria) using Orbitrap-based HRMS workflows. Implementing orthogonal chromatographic separations, the selection of stationary phases, and gradient conditions provided maximum selectivity and coverage of the benchmark metabolome at reasonable throughput. The integrated RP-LC and HILIC separations reflect the current state of LC–MS-based metabolomics and lipidomics. The library assessment comprised orthogonal column chemistries, which are reversed phase-liquid chromatography (RP-LC) and hydrophilic interaction liquid chromatographic (HILIC) separation at different pH values (low and high) using positive and negative electrospray ionization. Typically, these workflows enable the annotation of metabolites at concentrations ranging from sub-nM to µM. Compound identification was based on accurate mass (+/− 5 ppm), matching retention times, and MS/MS spectra as compared to authentic standards and internal databases built upon these standards. While the major body of the library was obtained upon analysis of an aqueous dilution by the streamlined combination of HILIC-HRMS (at pH = 8.0–9.0) and RP-LC-HRMS (at pH = 2.0) (raw data MTBLS1782), some compounds, such as lipids, carnitines, and coenzymes required alternative sample reconstitution/preparation and dedicated chromatographic separation (Supporting Information, material and methods part, Targeted metabolomics of interesting metabolite classes). More specifically, for coenzyme and acyl-carnitines analysis, a dedicated RP-LC-HRMS method utilizing ammonium bicarbonate at neutral pH [39] was implemented. Coenzyme A, acetyl coenzyme A, L-carnitine, O-acetyl-L-carnitine, palmitoyl-L-carnitine, and propionyl-L-carnitine could be annotated. For lipid analysis, the dry ethanolic extract was reconstituted in 50% ACN and measured using RP-LC-HRMS with a gradient involving IPA. As a result, 27 consistently (different batch, different instruments) identified (accurate mass, retention time, and MS/MS comparison to a multi-lipid standard mix) phospholipids from the classes PC, PE, PG and PS (LPC 16:0, LPC 18:0, LPC 18:1, PC 34:0, PC 34:1, PC 34:2, PC 34:3, PC 34:4, PC 36:2, PC 36:3, PC 36:4, PC 36:5, PC 36:6, PE 34:1, PE 34:2, PE 34:3, PE 36:2, PE 36:3, PE 36:4, PE 36:5, PG 34:0, PG 36:0, PS 34:1, PS 34:2, PS 34:3, PS 36:2, PS 36:3) were recovered.

**Figure 1:**
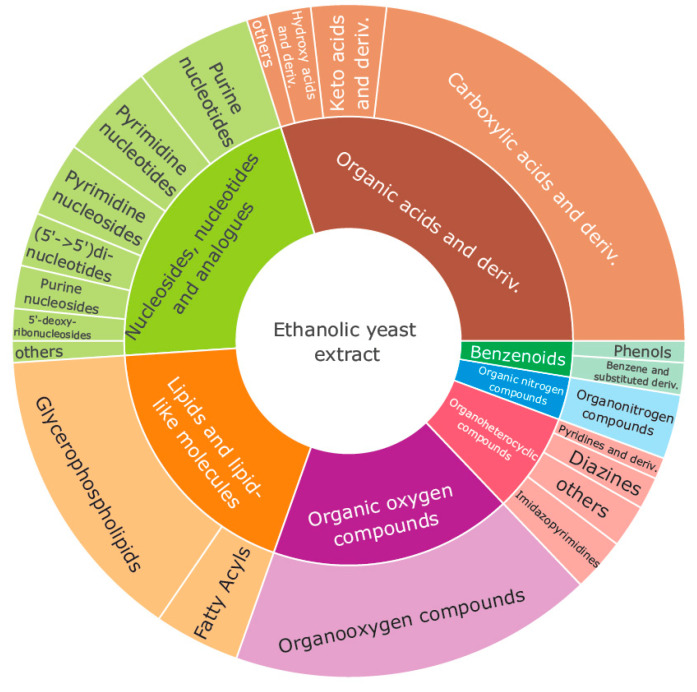
Annotated metabolite classes in ethanolic yeast extract using the classyFire [40] annotation system.

Literature search confirmed our findings and additionally expanded the reported compounds [27,28,29,30,31,32,33,34,35,36] in our library, which can be found in the Supplementary Table S1 (Excel sheet FinalMetaboliteList). As yeast is a eukaryotic organism, key regulatory elements are highly conserved between yeast and humans [19]. The curated list includes 206 metabolites and lipids (expected, detected, quantified in HMDB) present in the ethanolic yeast extract using orthogonal RP-LC-HRMS and HILIC-HRMS workflows, of which 181 metabolites were reproducibly identified (at least in two different fermentation batches, different instruments, two participating laboratories or others [27,28,29,30,31,32,33,34,35,36]). The additional 25 metabolites were reported elsewhere [27,28,29,30,33,34] (Supplementary Table S1, column, FinalMetaboliteList, “Additional literature hits”). Figure 1 shows that the 206 metabolites represent a huge range of different metabolites from the superclasses of (1) organic acids and derivatives making up 27% and the highest number of identifications (62 IDs) in the presented metabolite, followed by (2) nucleosides, nucleotides, and analogs (43 IDs), (3) organic oxygen compounds (37 IDs), (4) lipids and lipid-like molecules (36 IDs), (5) organoheterocyclic compounds (16), (6) organic nitrogen compounds (7 IDs) and (7) benzoids (5 IDs). The classification in Figure 1 is based on the classyFire system [40], categorizing in kingdoms, superclasses, classes, subclasses, and molecular frameworks. All 206 metabolites are level 1 identified corresponding to A-C of the newly proposed definition by the metabolomics society (e.g., D-sedoheptulose 7-phosphate) or known carbon, fatty acid identity, and double bond number for the identified lipids (e.g., PE(18:1_18:3)) [41,42].

The human metabolome database (HMDB 4.0) [43,44] was interrogated regarding overlaps with the yeast metabolome database (YMDB), which is based on the baker’s/brewer’s yeast *Saccharomyces cerevisiae* [45] crosschecking our library for plausibility. Most metabolites were present in the YMDB 2.0 [45] (170 from 206, accessed 15 July 2020), and all metabolites were present in the HDMB [44] (Supplementary Table S1). The higher match of our internal *Pichia pastoris* yeast extract database with the HMDB compared to YMDB can be explained by a more frequent comprehensive database curation of the HMDB (e.g., additional “status” ontology in HMDB, including expected metabolites) and by the fact that the YMDB is based on the different yeast strain *S. cerevisiae*. The high coverage of the HDMB and the YMDB, as well as the presence of several human key pathways, proves the potential of the proposed benchmark material. The related pathways playing a critical role in health and diseases are provided in Supplementary Table S1 (FinalMetaboliteList, column “pathway_names”). One hundred metabolites were reported to be involved in cancer, such as glucose (d-glucose, 6-phosphogluconic acid, gluconic acid, N-acetyl-D-glucosamine) and amino acids (l-arginine, l-asparagine, l-aspartic acid, l-cysteine, l-cystine, l-glutamic acid, l-glutamine, l-histidine, l-isoleucine, l-lactic acid, l-leucine, l-lysine, l-malic acid, l-methionine, l-phenylalanine, l-proline, l-serine, l-threonine, l-tryptophan, l-tyrosine, l-valine), which are uptake related compounds. These are known to be hallmarks of cancer metabolism [46]. Another example are obesity-related pathways with a range of involved lipids (LysoPC(18:0), LysoPC(18:1). PC(16:0_18:1), PC(16:0_18:2), PC(16:0_18:3), PC(16:0_20:4), PC(18:0_18:3), PC(18:2_18:3), PC(18:3_16:1), PC(18:3_18:3)) and lipid precursors (l-carnitine, l-acetylcarnitine, l-palmitoylcarnitine, propionyl-l-carnitine), known to trigger obesity formation upon general lipid accumulation [47].

Similar classes and metabolite numbers (ca. 200) are covered compared to a recent study on large-scale metabolomics using three different human plasma reference materials [12]. However, such plasma reference materials are expensive, have to be pooled from several hundred individuals, and characterized prior to their use as quality control. Hence, they cannot be produced in the exact same way as it is possible using a stable fermenter and the same yeast strain. The general availability of the yeast material enables us to further expand the library on a regular basis by identifying metabolites with new commercial standards. It must be emphasized that the given *Pichia pastoris* inventory is restricted to the implemented biomass preparation resulting from fermentation conditions, the number of cells submitted to extraction, and the ethanolic extraction procedure itself. The panel can be significantly changed upon using different extractions. The use of methanol/chloroform (Folch) extraction [48], for example, expanded the lipid panel with regard to nonpolar lipids, such as triglycerides, diglycerides, ceramides or ergosterol [21,49,50].

#### 2.1.2. Metabolite Stability and Yeast Fermentation Reproducibility

Benchmarking can be performed at different levels, either for checking instrumental/method performance in general (e.g., upon introduction of new MS instrumentation) or for daily quality control. In principle, the complete library is available for performance tests provided that the yeast-derived standard is stable under defined storage conditions. In order to prove the suitability of the proposed benchmark, NTA by RP-LC/HILIC-HRMS was performed of aliquots (*n* = 3) of ethanolic extracts from individually fermented batches (3 fermentations performed in 2017, 2018, 2019, respectively), which had been stored at −80 °C. The streamlined workflow (HILIC at pH 8.0, RP-LC at pH 2.0) obtained from one laboratory (Vienna BioCenter Core Facilities, Vienna, Austria) within the same sequence revealed a list of 151 compounds (see Supplementary Table S1, Batch comparison). These 151 compounds corresponded to 104 unique metabolites and were present in all three batches as well as the pooled control with excellent mass accuracy and retention time stability (ppm error: between −2.6 ppm and +0.5 ppm, RT error ± 0.4 min, Supplementary Table S1, Batch comparison) derived by HILIC- or RP-MS analysis. Figure A2A,B reveal clustering of the different batch fermentation replicates (*n* = 3) and separation by batch year (2017, 2018, 2019). Due to batch clustering and randomly assigned samples, this observation proves stable chromatographic conditions for both HILIC and RP. As the 151 confirmed compounds were consistently found in all batches and aliquot samples (Supplementary Table S1, Batch comparison), stability over the course of three years was proven when stored at −80 °C. A low concentration of a metabolite might be responsible for compounds with elevated group coefficients of variation (CVs) or through inappropriate integration of the software and are marked with an asterisk. Another way of visualizing sample comparability is box-and-whiskers plots showing data distribution (Figure A3A,B). Overall, good repeatability through all yeast fermentation samples and quality controls was observed. Intrabatch repeatability was further monitored, comparing the different aliquots of one yeast fermentation (*n* = 3 per batch). Excellent average group CVs of 9–13% were observed for 126 compounds (see Supplementary Table S1, BC-filtered ratios 0.5 to 5). These 126 compounds (pH ILIC-MS: 76 metabolites, RP-MS: 50 metabolites; filter: response ratios between 0.5 and 5 was set as criterion leading to average group CVs lower than 15%) were reproducibly identified by accurate mass, matching retention times, and MS/MS (Supplementary Table S1, BC-filtered ratios 0.5 to 5). As stability over the years is important for suitable benchmarking material, we further elucidated compound stability and inter-batch/batch-to-batch reproducibility. Overall, 67 metabolites were identified with comparably stable areas over all three yeast fermentations (Figure A4) performed within three years (batch 2017, 2018, 2019). Hence, these 67 metabolites are the best choice of our metabolite panel for benchmarking non-targeted metabolomics datasets and are highlighted green in Supplementary Table S1 (BC-filtered ratios 0.5 to 5). In the case that a metabolite was detected with both methods, the more suitable method was chosen considering retention time and sensitivity. An additional 14 metabolites were found at similar excellent CVs but could not be annotated at the highest (level 1) confidence. Despite matching with the commercial standard in terms of accurate mass and retention time, MS/MS information was either not sufficient or not available (Supplementary Table S1, BC-filtered ratios 0.5 to 5). Overall, *Pichia pastoris* yeast extract is an excellent source to perform quality controls in non-targeted metabolomics experiments as reproducible and low-cost production in high amounts is possible, yielding an interesting panel of essential eukaryotic metabolites and lipids. As commonly done in proteomics workflows using protein identification (IDs) numbers in HeLa quality controls, monitoring the number of metabolite IDs in the yeast extracts can be used to judge instrument or method performance.

### 2.2. Application of the Benchmark Material for Non-Targeted Metabolomics

#### 2.2.1. Yeast Quality Controls for Instrument Performance

**Figure 2:**
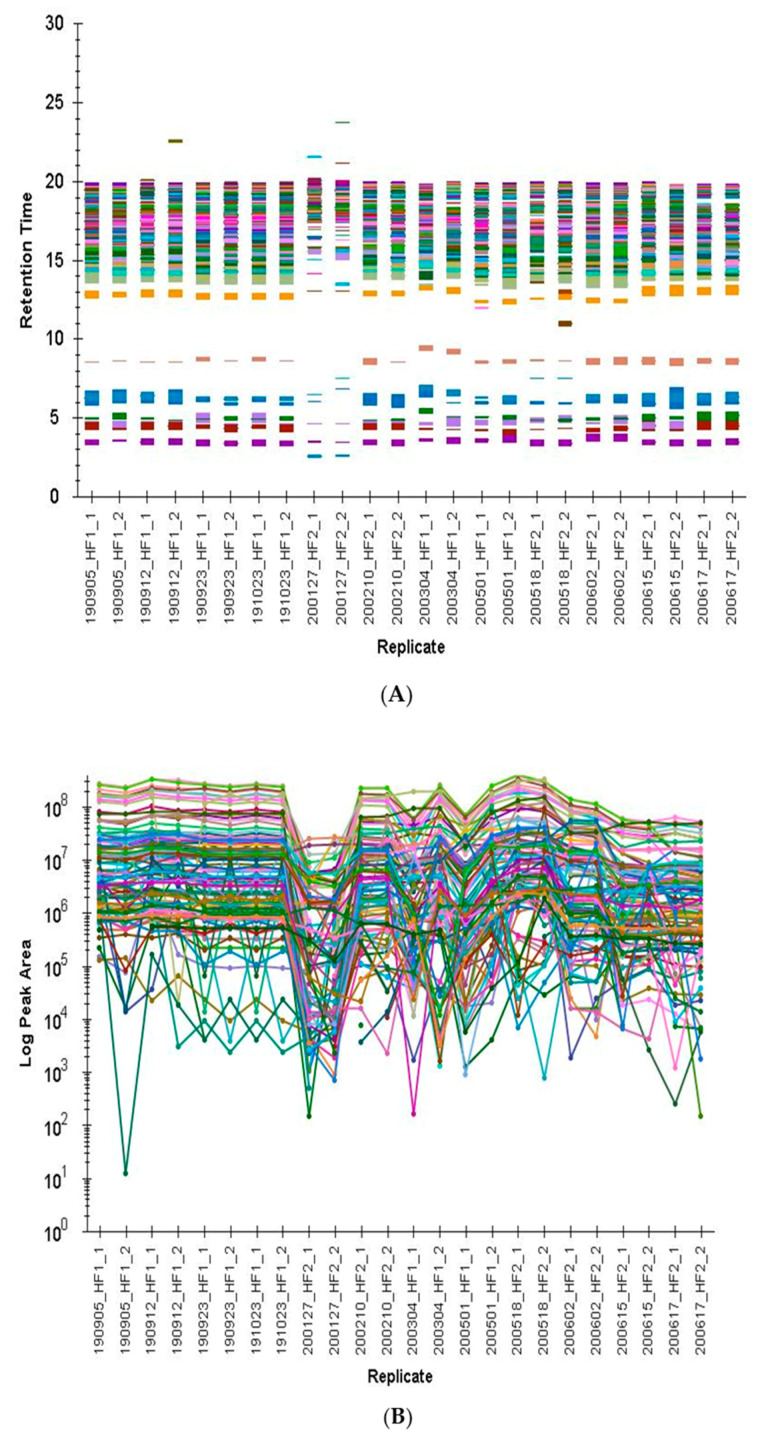
(**A**) Yeast extract retention time stability of the reversed-phase high-resolution mass spectrometry (RP-HRMS) targeted lipid method on two different high-resolution mass spectrometers (Orbitrap HF 1, HF 2) over the time course of 10 months using Skyline for visualization [51]. (**B**) Yeast extract control of 26 lipids using RP-HRMS to monitor the performance of two Orbitrap mass analyzers (HF 1, HF 2) using Skyline for visualization [51].

The obtained biological repeatability of the fully controlled fermentations, together with the storage stability of the dried extraction aliquots over three years, makes the yeast benchmark fit for purpose for a whole range of metabolomics measurement platforms. Figure 2A,B show the application of the yeast benchmark as our in-house quality check routine of a lipidomics method. 26 lipids were analyzed by RP-LC-HRMS (LPC 16:0, LPC 18:0, PC 34:0, PC 34:1, PC 34:2, PC 34:3, PC 34:4, PC 36:2, PC 36:3, PC 36:4, PC 36:5, PC 36:6, PE 34:1, PE 34:2, PE 34:3, PE 36:2, PE 36:3, PE 36:4, PE 36:5, PG 34:0, PG 36:0, PS 34:1, PS 34:2, PS 34:3, PS 36:2, PS 36:3) on two instruments (Q Exactive™ HF Hybrid Quadrupole-Orbitraps, instrument HF 1 and HF 2) and are plotted with respect to peak area and retention time in order to control the instrumental performance prior measurement sequences. The measurement from 27 January 2020 (200127_HF2) showed a significant signal decrease requiring cleaning of the instrument (S-lens, shield cleaning). The signal improved as validated with the in-house yeast lipid quality control from February 2020 (Figure 2B, 2002010_HF2_1 and 20200210_HF2_2). Not considering measurement outliers (i.e., 200127_HF2 replicate 1 and 2), the retention time repeatability over the course of 10 months for each instrument was excellent, with most of the lipids (19 out of 26 lipids) having a variation of <5% (ranging from 0.4% for high abundant PE 34:1 with areas of 10^6^ to 10% for low abundant PI 34:3 with areas of 10^3^). Figure 2A shows all lipids, while Figure A5 depicts the biggest lipid class of PC for further insight. The overall plotted peak areas cover 5 orders of magnitudes, resembling the typical situation in metabolomics type of measurements (Figure 2B).

#### 2.2.2. Yeast Quality Controls Facilitating Method Development

No single method or platform can cover the full metabolome due to the polarity range of metabolites (from highly water-soluble sugars to very lipophilic triacylglycerols) [52]. Benchmarking materials by providing a defined panel of metabolites in a biological matrix can facilitate the development and proper validation of mass spectrometry-based assays. A metric is introduced, allowing the evaluation of the metabolome coverage with regard to other platforms/procedures. In our laboratory, we successfully applied endogenous and labeled yeast extracts for method validation of different LC-MS based workflows, such as (1) merged metabolomics and lipidomics workflow based on the combination of HILIC and RP [24], (2) anion exchange chromatography coupled to high-resolution MS [20], (3) polar and nonpolar lipid analysis by online HILIC and RP combination [53], and (4) comparison of different LC–MS-based workflows [37,54]. In this work, high metabolite coverage of ethanolic yeast extracts was achieved using non-targeted orthogonal HILIC and RP-MS (Supplementary Table S1, Batch comparison). To extend the metabolome coverage of the benchmarking material, additional LC–MS methods were developed to target (1) acylcarnitines and coenzymes (lower abundance and problems with ionization efficiency using standard metabolomics workflows) and (2) lipids (different solubility). Using the targeted neutral RP method, coenzyme A, acetyl coenzyme A, L-carnitine, O-acetyl-L-carnitine, palmitoyl-L-carnitine, and propionyl-L-carnitine were identified (MS/MS matching to commercial standards, *m*/*z*, RT; exemplary shown for O-acetyl-L-carnitine, Figure A6) and led to estimated concentrations (comparison to 5 µM standard mix) in the low to high nM range (Table A1). The dedicated lipid analysis led to the identification of 26 additional phospholipids as described above to embrace the broad diversity of the metabolome (Figure 2A).

These results show the high metabolome coverage of the yeast material, which can be interrogated to develop dedicated LC–MS methods for specific metabolite classes. Therefore, yeast ethanolic extracts are an ideal test matrix to establish new metabolomics workflows.

## 3. Discussion

In the last years, yeast ethanolic extracts were extensively used in our laboratory produced by the described controlled *Pichia pastoris* fermentation. We found that the material provides a sufficiently large coverage of the eukaryotic metabolome. Our reproducible metabolite database was derived from different fermentation batches, which were measured in two different laboratories. Additional metabolites were incorporated into the library that was identified in the literature and measured by groups working with this yeast material, which is also commercially available and internationally distributed (300 $ for the unlabeled yeast, 500 $ for the ^13^C-labeled yeast yielding ca. 50–100 sample aliquots). The provided list of 206 metabolites covers the classes of (1) organic acids and derivatives (2) nucleosides, nucleotides, and analogs, (3) lipids and lipid-like molecules, (4) organic oxygen compounds, (5) organoheterocyclic compounds, (6) organic nitrogen compounds, and (7) benzoids and can be further extended using commercially available standards and different extraction strategies. All yeast metabolites were also reported in the human metabolome database, and 104 out of 206 metabolites were stable for several years when stored at −80 °C. Out of these 104 compounds, 67 were found to be the most stable and are the ideal starting point for benchmarking experiments, such as method development or instrumental performance tests.

Commercially available materials, such as human plasma reference materials (e.g., SRM 1950 or CHEAR), are expensive. As they must be pooled from several hundred individuals and characterized prior to their use as quality control, reference materials from human origin can never be reproduced in the exact same way. The yeast ethanolic extract is of eukaryotic origin, easily accessible, commercially available, and can be produced under controlled fermentation conditions. Hence, the yeast material is a perfect long-term, low-cost alternative as it can be reproduced/ordered whenever necessary. This means, yeast extracts are an ideal starting point for broader community-wide used NTA quality controls. Hence, by using the number of identified metabolites in the yeast extract, a transfer of the proteomics quality control strategy based on HeLa cell extracts is possible in the metabolomics context. Expansion of the database on a regular basis in our laboratory, as well as further extensive use by other metabolomics laboratories [27,28,29,30,33,34], will lead to a deep characterization of these extracts and a very detailed list of the contained metabolites. Moreover, batch normalization is feasible with ethanolic yeast extracts. Hence, we dare to propose the described yeast material as an ideal open-source database to benchmark the coverage of non-targeted metabolomics workflows.

## 4. Materials and Methods

### 4.1. Standards and Solvents

Metabolite standards were purchased from Sigma-Aldrich (Vienna, Austria) or Carbosynth (Berkshire, UK). Lipid Standards were obtained from Avanti Polar Lipids, Inc. (Alabaster, AL, USA), Sigma-Aldrich (Vienna, Austria) or Carbosynth (Berkshire, UK). All lipid and metabolite standards were weighed, dissolved in an appropriate solvent (mostly methanol or water), and a multi-metabolite, as well as a multi-lipid mix, were prepared. The standard reference material (SRM) 1950 metabolites in frozen human plasma was purchased from the National Institute of Standards and Technology (NIST) (Gaithersburg, USA). Acetonitrile (ACN), isopropanol (IPA), methanol (MeOH), and water were of LC–MS grade and ordered from Fisher Scientific (Vienna, Austria) or Sigma-Aldrich (Vienna, Austria). Ammonium bicarbonate, ammonium formate, and ammonium hydroxide were ordered as LC–MS grade eluent additives from Sigma-Aldrich. Formic acid was also of LC–MS grade and obtained from VWR International (Vienna, Austria).

### 4.2. Production of Ethanolic Yeast Extracts

*Pichia pastoris* (Guillierm.) Phaff 1956 (*Komagataella phaffii Kurtzman*) [38] was cultivated in a New Brunswick BioFlo 310 fed-batch fermenter for 38 h (Eppendorf, Hamburg, Germany) with full control over the input variables in terms of glucose as carbon source (Cambridge Isotope Laboratories, Tewksbury, MA, USA), pH, temperature, and oxygenation applying an adapted protocol for the production of ^13^C-labeled yeast extracts [22]. Process monitoring was facilitated by online measurement of pH, temperature, and dissolved oxygen. Offline assessment of optical density at 600 nm (OD_600_) and optical cell counting was performed at several time points. The cells were fermented until an OD_600_ of 12. At the end of the process, the biomass was quenched in 60% methanol (*v*/*v*) at −30 °C and subsequently extracted in boiling 80% ethanol (*v*/*v*) for metabolites. Finally, the ethanolic extract was aliquoted and dried in a vacuum centrifuge (Genevac EZ2, Ipswich, United Kingdom). Aliquots of the *Pichia pastoris* ethanolic extracts, derived from ~2 billion yeast cells (corresponding to 20 mg of dry cell weight), were dried in 15 mL Falcon tubes and stored as a pellet at −80 °C prior to analysis. As a commercially available alternative, the unlabeled yeast extract can be ordered via ISOtopic solutions (Vienna, Austria) or its global distributor, Cambridge Isotope Laboratories (Tewksbury, MA, USA).

### 4.3. LC–MS Analysis and Data Analysis of Yeast Extracts

#### Untargeted Metabolomics

**Figure A1:**
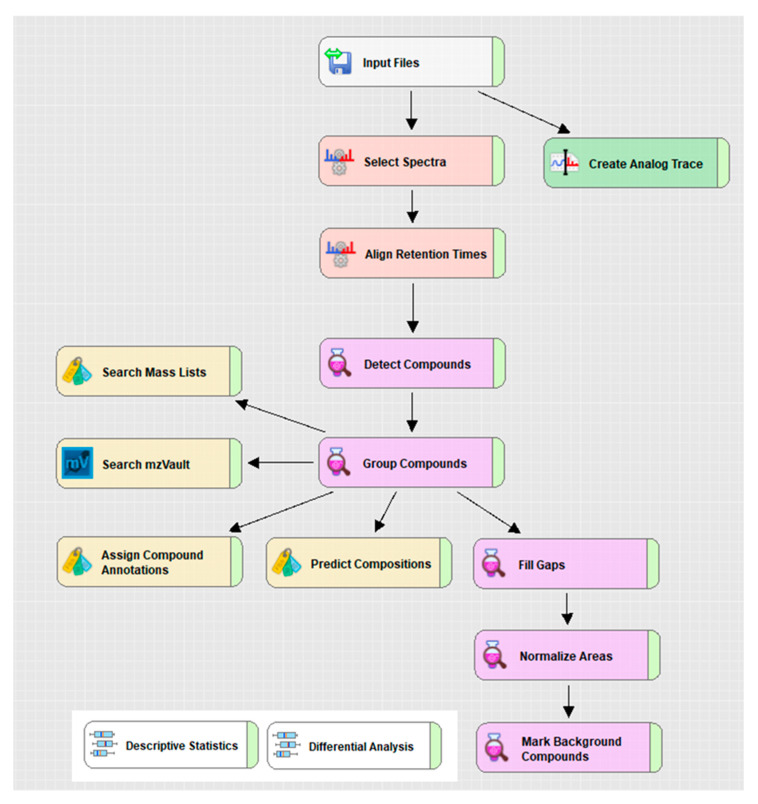
Compound Discoverer 3.1.0.305 workflow for the untargeted reversed-phase (RP) and hydrophilic interaction liquid chromatographic (HILIC-MS) analysis. Detailed parameters can be found here: www.ebi.ac.uk/metabolights/MTBLS1782 (access on 8 March 2021).

**Figure A2:**
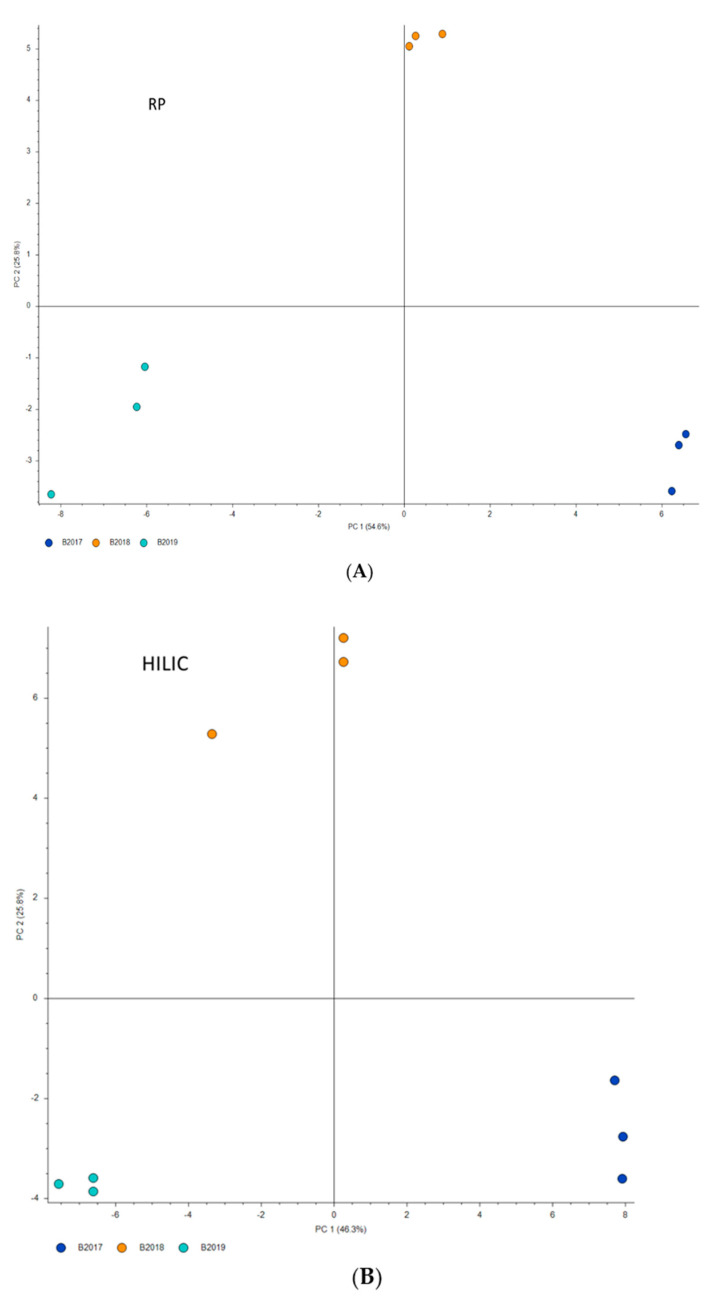
(**A**) Principal component analysis (analyzed in Compound Discoverer) of all identified metabolites in different batches (2017, 2018, 2019, stored at −80 °C until analyzed) of RP-HRMS data; (**B**) principal component analysis (analyzed in Compound Discoverer) of all identified metabolites in different batches (2017, 2018, 2019, stored at −80 °C until analyzed) of HILIC-HRMS data.

**Figure A3:**
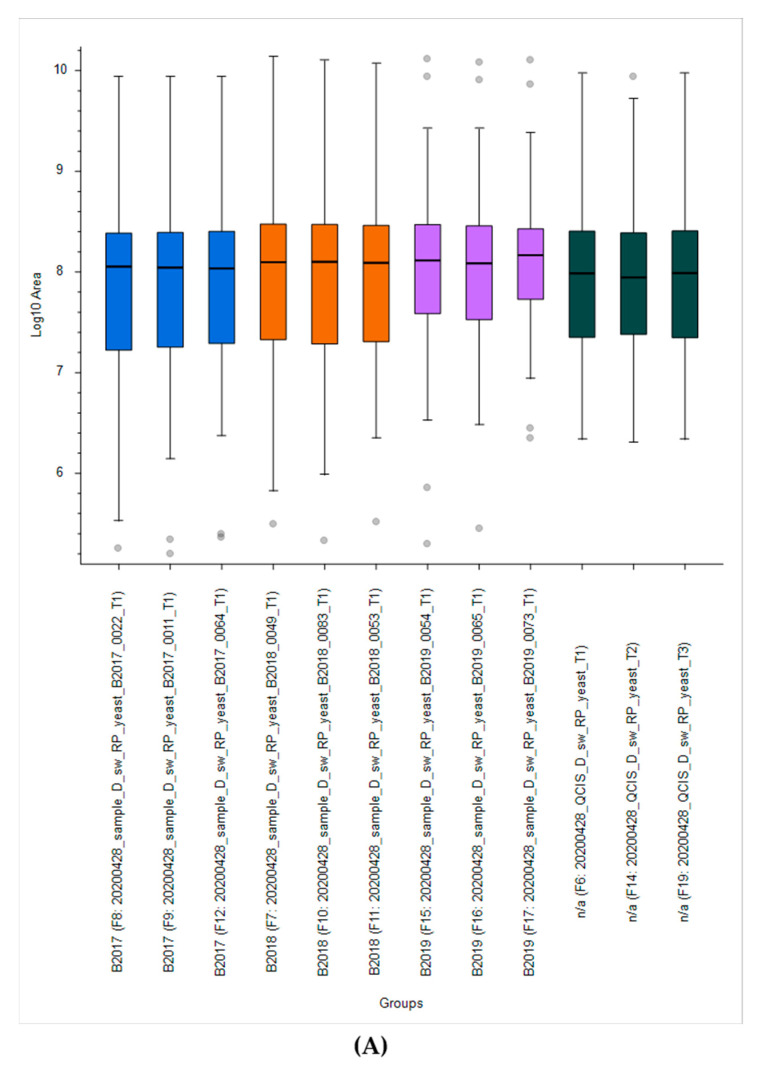

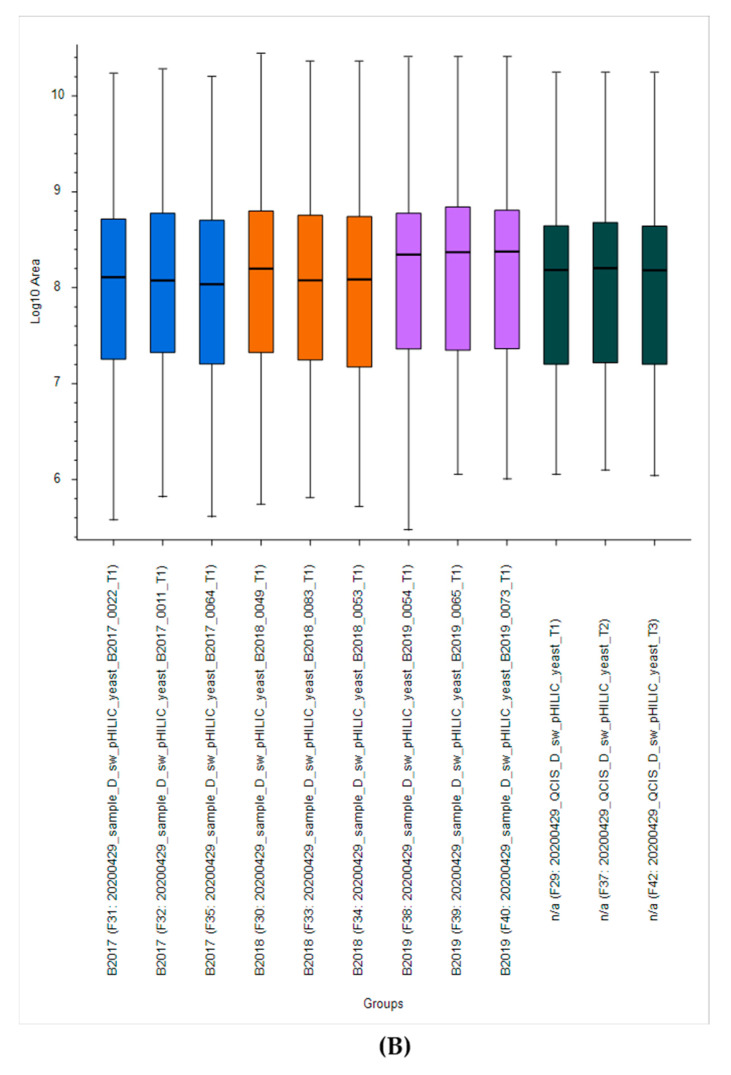
(**A**) Box-and-whisker plot of the data distribution. Each box corresponds to the annotated metabolite areas of each sample on a logarithmic scale (log_10_) measured with RP-HRMS. Ethanolic yeast extracts (*n* = 3) from three different years (2017, 2018, 2019) and the mixed quality control (*n* = 3) are compared. Variance depicts the broad concentration range of the metabolome. Data were analyzed using Thermo Compound Discoverer. (**B**) Box-and-whisker plot of the data distribution. Each box corresponds to the annotated metabolite areas of each sample on a logarithmic scale (log_10_) measured with HILIC-HRMS. Ethanolic yeast extracts (*n* = 3) from three different years (2017, 2018, 2019) and the mixed quality control (*n* = 3) are compared. Variance depicts the broad concentration range of the metabolome. Data were analyzed using Thermo Compound Discoverer.

**Figure A4:**
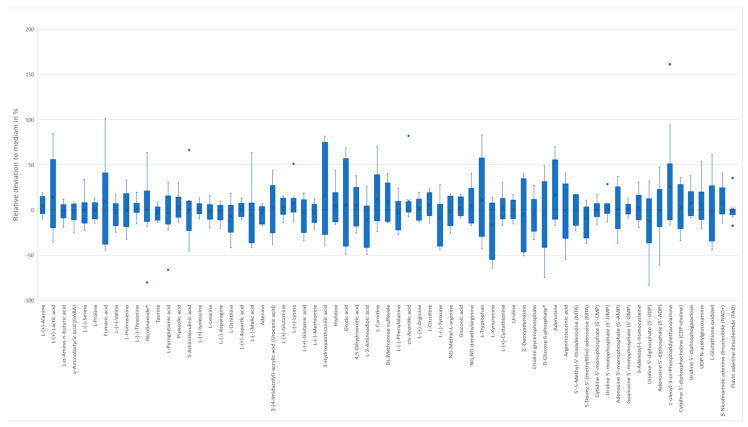
Relative deviation of all the samples to the median sample area of the 67 most stable compounds (more information on the stable metabolites can be found in Supplementary Table S1, BC-filtered ratio). Addition of (+) and (-) indicates the polarity of measured compounds, if no labeling is assigned, both modes can be used.

**Figure A5:**
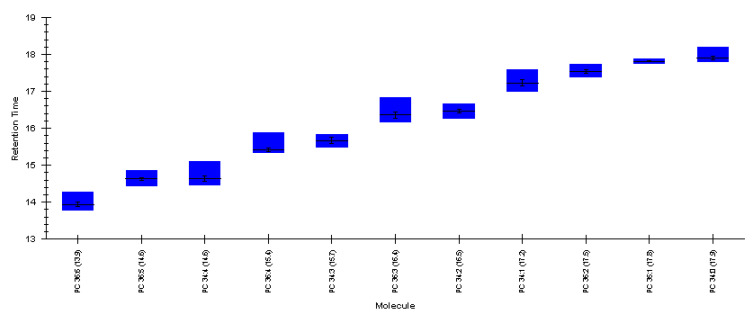
Retention time stability of the exemplary class of phosphatidylcholines (PCs) in the yeast control followed over the time course of 10 months.

**Figure A6:**
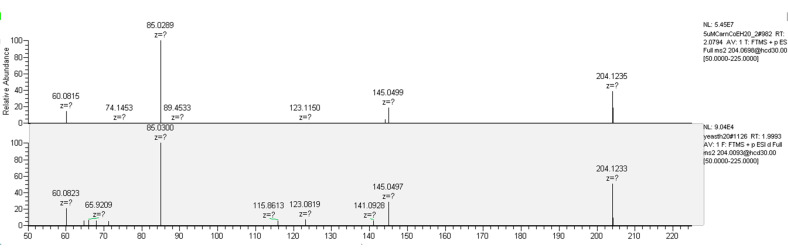
Fragmentation spectra of O-acetyl-L-carnitine in 5 µM standard and ethanolic yeast extracts measured by neutral RP-HRMS in positive mode.

Samples of dried ethanolic yeast pellets (derived from two different batches: November 2017, May 2018) were dissolved in 2.5 mL MilliQ water (Advantage Q10, Merck, Darmstadt, Germany), and sample batch May 2019 was filled up to a volume of 5 mL with MilliQ water. An aliquot of 100 µL was transferred into an Eppendorf tube and centrifuged at 4 °C for 10 min at 15,000 RCF. 50 µL of the supernatant was diluted 1:1 (*v*/*v*) with acetonitrile or 0.1% formic acid for HILIC or RPmeasurements, respectively. Ten microliters of each sample was pooled and used as a quality control (QC) sample. Samples were randomly assigned into the autosampler, and metabolites were separated on a SeQuant ZIC-pH ILIC HPLC column (Merck, 100 × 2.1 mm; 5 µm with guard column) or an RP-column (Waters, ACQUITY UPLC HSS T3 150 × 2.1 mm; 1.8 μm with VanGuard column) with a flow rate of 100 µL min^−1^ delivered through an Ultimate 3000 HPLC system (Thermo Fisher Scientific, Germany). The stepwise gradient for HILIC analysis (adapted from Wernisch and Pennathur [55]) involved starting conditions of 90% A (100% ACN), ramp to 25% B (25 mM ammonium hydrogen carbonate, pH 8) within 6 min, 2 min hold at 25% B, from 8 to 21 min a ramp to 60% B was applied, switching to 80% B at 21.5 min followed by a flushing (21.5–26 min: 80% B) and re-equilibration step (26.1–35 at 10% B). The gradient for RPLC analysis involved a linear ramp-up time of 20 min starting with 99% A (0.1% formic acid) to 60% B (0.1% formic acid in ACN) followed by 5 min hold (21–26 min) at 90% B and a re-equilibration step (26.1–36 min: 1% B). All samples were analyzed by both HILIC and RP separation followed by ESI-HRMS in polarity switching mode applying the mass range of 70–900 *m*/*z* at a resolution of 70,000 *m*/*z* (12 Hz). MS/MS spectra were acquired by data-dependent high-resolution tandem mass spectrometry at 17,500 resolution with normalized collision energies of 25 (a.u.) on a Q Exactive Focus (Thermo Fisher Scientific, Bremen, Germany). Ionization potential was set to +3.5/−3.0 kV, the sheath gas flow was set to 20, and an auxiliary gas flow of 5 was used. Samples were analyzed in a randomized fashion bracketed by a blank and pooled QC sample for background correction and normalization of the data, respectively. QC samples were additionally measured in confirmation and discovery mode to obtain further MS/MS spectra for identification. Obtained data sets were processed by compound discoverer (CD) 3.1.0.035 (Thermo Fisher Scientific). A detailed description of the CD nodes and parameters can be found in the supporting information (Figure A1) and in the protocols section of the project MTBLS1782 in MetaboLights [11]. Compounds were annotated by comparing the retention time against our internal mass list database, which was generated with authentic standard solutions. Ordering numbers and MS/MS match scores can be found in Supplementary Table S1 (Tab Batch comparison). A retention time window of 0.4 min and mass accuracy of 5 ppm for precursor masses were set. In addition, MS/MS spectra were compared against our internal MS/MS database taking 10 ppm for fragment ion masses into account as well. MS/MS match scores were derived from our internal database by using the mzVault node in the compound discoverer. HILIC-MS and RP-MS raw data file can be found here: www.ebi.ac.uk/metabolites/MTBLS1782 (accessed on 8 March 2021).

### 4.4. Targeted Metabolomics of Interesting Metabolite Classes

#### 4.4.1. Lipids

The dried yeast extract was dissolved in 2 mL 50% acetonitrile for lipid analysis using reversed-phase separation with an isopropanol gradient. The chromatographic separation was performed using an Acquity HSS T3 (2.1 mm × 150 mm, 1.8 μm, Waters, Etten-Leur, Netherlands) with a VanGuard pre-column. Solvent A was ACN/H_2_O (3:2, *v*/*v*), and solvent B was IPA/ACN (9:1, *v*/*v*); both solvents contained 0.1% formic acid and 10 mM ammonium formate. The following gradient was applied: 0–2 min: 30% B, 2–15 min: ramp to 75% B, 15–17 min: to 100% B, 17–22 min: holding 100% B, 22–27 min: 30% B. The injector needle was washed with 75% IPA, 24.9% H_2_O, and 0.1% formic acid prior to each injection. High-resolution MS with a Q Exactive HF (Thermo Fisher Scientific) was performed using ddMS2 (Top10) for lipid detection and the following heated electrospray ionization (HESI)source parameters: capillary temperature of 270 °C, sheath gas flow rate of 50, an auxiliary flow rate of 14, sweep gas of 3, S-lens RF level of 45 and auxiliary gas heater temperature of 380 °C applying a spray voltage of 3.5 kV in positive mode and 2.8 kV in negative mode Skyline (Version 20.1.0) was used to create MS1 area-based quality control charts [54].

#### 4.4.2. Carnitines and Coenzymes

The dried yeast extract was dissolved in 2 mL 100% water for carnitines and coenzymes analysis by reversed-phase separation adapted from Neubauer et al. [39]. The chromatographic separation was performed using an Acquity HSS T3 with pre-column (as stated for the lipids). Solvent A was 50 mM NH_4_HCO_3_, pH 6.94, and solvent B was 100% ACN, and the following gradient was applied: 0–7 min: ramp from 100% A to 20% B, 7–8.1 min: ramp to 40% B, 8.1–10 min: 40% B, 10.1–13 min: 100% A. MS detection was performed by Q Exactive HF (Thermo Fisher Scientific, Bremen, Germany) using positive ionization mode (+3.5 kV), S-lens RF level of 30 and ddMS2 (Top10) for carnitine and coenzyme analysis.

#### 4.4.3. Statistical Analysis

Statistical data evaluation, including PCA, descriptive analysis (box plots), p-values, adjusted *p*-values, log2-fold change and group CVs, were performed with compound discoverer (CD) 3.1.0.035 (Thermo Fisher Scientific, Bremen, Germany). The most stable compounds were determined using filtered ratios (0.5–5, Supplementary Table S1) of the batch comparison, and their reproducibility was investigated by box blots in Microsoft Excel 365.

## Data Availability

HILIC-MS and RP-MS raw data files of the ethanolic yeast extracts are available here: www.ebi.ac.uk/metabolights/MTBLS1782.

## Supplementary Materials

The following are available online at https://www.mdpi.com/2218-1989/11/3/160/s1, Table S1: (1) list of metabolites and lipids identified in ethanol extract, (2) batch comparison (2017/2018/2019), (3) group area batch comparison ratios filtered.

## Appendix A

This section contains the extended methods section and additional information on materials and methods, (Figure A1) compound Discoverer 3.1.0.305 workflow, (Figure A2) RP-HRMS and HILIC-HRMS batch comparison of the metabolites areas derived from the compound discoverer, (Figure A3) principal component analysis of the identified metabolites of three different yeast batches by HILIC- and RP-MS/MS, (Figure A4) relative deviation of all the samples to the median sample area of the 67 most stable compounds, (Figure A5) retention time stability of the yeast control in the exemplary class of PCs, (Figure A6) MS2 of O-acetyl-L-carnitine in 5 µM standard and ethanolic yeast extracts, and (Table A1) estimated carnitine and coenzyme concentrations in ethanolic yeast extract.

**Table A1:** Estimated carnitine and coenzyme concentrations in ethanolic yeast extract determined using neutral RP-HRMS and 5 µM commercially available standards.

		M + H	RT	Est. Concentration (nM)
Coenzyme A	C_21_H_36_N_7_O_16_P_3_S	768.1225	6.29	2792
Acetyl coenzyme A	C_23_H_38_N_7_O_17_P_3_S	810.1330	6.60	316
Palmitoyl coenzyme A	C_37_H_66_N_7_O_17_P^3^S	1006.3522	7.10	<LOQ
Carnitine	C_7_H_15_NO_3_	162.1125	1.48	139
O-acetyl-L-carnitine	C_9_H_17_NO_4_	204.1230	2.16	16
Propionyl-L-carnitine	C_10_H_19_NO_4_	218.1387	3.79	3
